# Free muscle transfer for challenging and rare upper limb disorders

**DOI:** 10.1186/1753-6561-9-S3-A56

**Published:** 2015-05-19

**Authors:** Kazuteru Doi

**Affiliations:** 1 Department of Orthopaedic Surgery, Ogori Daiichi General Hospital, Ogori, Yamaguchi, 754-0002, Japan

## 

Free innervated muscle transfer (FMT) achieves functional restoration following not only traumatic brachial plexus injury, but also intractable rare upper limb disorders, such Hopkins' syndrome, myoplasia in arthrogryposis and radition neuropathy. I introduce our current experience of FMT for these disorders.

Hopkins syndrome is a rare cause of poliomyelitis-like paralysis affecting one or more extremities after an acute attack of asthma. The exact etiology of Hopkins syndrome is not known. Two children developed acute asthma followed by complete flaccid paralysis of the the upper extremity. They underwent staged reconstruction using FMT. Rigorous postoperative physiotherapy was carried out to achieve a good functional outcome. At recent follow-up, they were able to effectively use the reconstructed hand for most of their daily activities. They had good control and could perform 2-handed activities. The selection of a suitable operative treatment and suitable donor nerves is critical, and there are no clear guidelines in the literature. FMT can be effectively employed in similar cases to restore grasping function.

Arthrogryposis is a collective term applied to a variety of different syndromes characterized by nonprogressive, multiple joint contractures present at birth due to neurogenic, myopathic (amyoplasia), or connective-tissue disorders. The most serious impairment of amyoplasia in the upper extremity is an inability to flex the elbow due to extension contracture of the elbow. The ability to flex one elbow is essential to enable a child to become functionally independent with self-feeding and self-care of the face and hair; extension of the other elbow is necessary for independent toileting. Active elbow flexion is the definitive goal of surgical intervention.

Multiple procedures, including triceps transfer, pectoralis major transfer, latissimus dorsi transfer, and the Steindler flexorplasty, have been used to provide active elbow flexion in patients with arthrogryposis. However, these conventional procedures do not always provide satisfactory long-term function and may result in a decreased arc of elbow flexion and in flexion contracture because of weakness of the donor muscles.

We present the long-term results of a functioning free muscle transfer with use of the gracilis muscle for restoration of elbow flexion in three patients with arthrogryposis.

Radiation-induced brachial plexopathy is a delayed complication of radiation treatment for tumors involving the neck and chest area and is progressive. A 56 year-old woman presented to us with loss of elbow flexion and weak wrist and finger extension 15 years after she received external beam radiation to the left chest, axilla, and supraclavicular region for the treatment of breast cancer. She was managed with a gracilis free muscle transfer for elbow flexion and hand prehension. By 2 years after surgery she regained elbow range of motion of 40 to 110 degrees and improved her hand function. She was able to perform her activities of daily living. Her Disabilities of the Arm, Shoulder, and Hand Score improved from 56 to 20.

